# The Methylation Status and Expression of Epstein-Barr Virus Early Genes BARF1 and BHRF1 in Epstein-Barr Virus-Associated Gastric Carcinomas

**DOI:** 10.1155/2017/3804146

**Published:** 2017-04-11

**Authors:** Jing Li, Wen Liu, Kui Che, Yan Zhang, Zhenzhen Zhao, Bing Luo

**Affiliations:** ^1^Department of Clinical Laboratory, The Affiliated Hospital of Qingdao University, 19 Jiangsu Road, Qingdao 266003, China; ^2^Department of Medical Microbiology, Qingdao University Medical College, 38 Dengzhou Road, Qingdao 266021, China; ^3^Department of Central Laboratory, The Affiliated Hospital of Qingdao University, 19 Jiangsu Road, Qingdao 266003, China; ^4^Department of Clinical Laboratory, Zibo Central Hospital, 19 Gongqingtuan Road, Zibo 255036, China

## Abstract

Epstein-Barr virus (EBV) is an important DNA virus which establishes latent infection in human malignancies. Expression of EBV-encoded genes in the associated tumors is strongly modulated by promoter CpG methylation of EBV genome. This study aimed to explore the methylation status of the promoters of EBV BamHI-A rightward frame 1 (BARF1) and BamHI-H rightward open reading frame 1 (BHRF1) and their influence on transcriptional expression, to further understand the roles of BARF1 and BHRF1 in the occurrence of EBV-associated cancer. We evaluated the methylation status of BARF1 and BHRF1 promoters in 43 EBV-associated gastric carcinoma (EBVaGC) tissues and EBV-positive cell lines. Their expressions were evaluated by real-time quantitative PCR. We found that the promoters of BARF1 and BHRF1 were methylated by varying degrees in different EBV-positive cell lines and were almost hypermethylated in all EBVaGC tissues. The methylation status of BARF1 and BHRF1 promoters were significantly reduced by 5-Aza-CdR along with the increasing gene expressions. Hypermethylation of Ap and Hp mediates the frequent silencing of BARF1 and BHRF1 in EBV-associated tumors, which could be reactivated by a demethylation agent, suggesting that promoter demethylation and activation is important for BARF1 and BHRF1 transcription and their further action.

## 1. Introduction

Epstein-Barr virus (EBV) is a gamma DNA herpes virus that infects over 95% of the global population and remains an asymptomatic life-long infection [1]. B lymphocytes and epithelial cells are the major targets of EBV infection. As a common human tumor virus, EBV was shown to be associated with a vast number of human diseases, such as lymphomas, nasopharyngeal carcinoma (NPC), and gastric carcinoma [2]. Epstein-Barr virus-associated gastric carcinoma (EBVaGC) is a distinct subtype that accounts for approximately 10% of all gastric carcinomas worldwide. EBVaGCs is formed by monoclonal proliferation of EBV-infected cell, and all carcinoma cells of EBVaGC are EBV-positive. Thus, EBV was suggested to be a causal role in gastric carcinogenesis, intimately linked to pathogenesis and tumor maintenance [3–5]. Numerous studies revealed that genomic features of host DNA, mRNA, microRNA, and CpG methylation profiles were contributed to the carcinogenesis of EBVaGCs [6, 7].

It is undoubted that EBV infection and the viral gene expression play contributory role in EBV-associated tumor pathogenesis. However, the detail mechanisms are still in question. Besides lytic replication, EBV infection was characterized to three distinct latency types: latency I, II, and III, by the expression patterns of EBV-encoded genes. The latency pattern in EBVaGCs belongs to either latency I or II, which expresses EBER, EBNA1, BART, LMP2A, and BART miRNAs [1, 4, 5]. The expression of viral genes varies depending on the tissue of origin and the state of the tumors [8, 9]. Furthermore, viral latent gene expression could also be suppressed by methylation. The viral gene methylation might be a response of the host cell against foreign DNA; on the other hand, it might benefit EBV by allowing it to escape the immune response of the host [5]. Epigenetic changes caused by DNA methylation play the most striking role in the tumorigenesis.

BamHI-A rightward open reading frame 1 (BARF1) and BamHI-H rightward open reading frame 1 (BHRF1) are two EBV early gene-encoding proteins homologous to human proto-oncogene c-fms and antiapoptotic gene Bcl-2, respectively. BARF1 expression is restricted to the viral lytic replication cycle in B cells and lymphomas [10, 11]. However, BARF1 was highly expressed in NPC and EBVaGC tissues in the absence of expression of lytic genes [12–15]. Thus, BARF1 is considered a viral oncogene in epithelial malignances and may play an important role in the development of NPC and gastric cancer [16]. Its oncogenic effect might transform epithelial cells and activate the expression of Bcl-2, enabling cell survival under inappropriate conditions [17]. The methylation status of the BARF1 gene promotor has been demonstrated in various cell lines in both epithelial carcinoma and B cell lines, and almost all CpGs were methylated. This indicates that transcription of BARF1 must overcome methylation-induced repression [15]. BHRF1 is homologous to human proto-oncogene Bcl-2, which is involved in the pathogenesis of a subset of B cell lymphomas. As a lytic gene, BHRF1 was originally thought to be expressed only in the virus lytic cycle. In EBV lytic stage, BHRF1 plays an important role in the effective replication of the virus and the release of mature viral particles. However, the BHRF1 expression was also reported in latent period in EBV-associated B cell lymphomas, NPC, and EBVaGC. The transcription of BHRF1 suggested a possible role in maintaining the persistent infection and pathogenesis in EBC-associated tumor [18–21].

In this study, we selected a series of EBV-positive cell lines derived from lymphocyte or epithelium and a number of EBVaGC tissues to study the promoter methylation status of EBV-encoded genes BARF1 and BHRF1 (Ap and Hp) and their effects on the expression of corresponding genes.

## 2. Material and Methods

### 2.1. Cell Lines and Tumor Samples

PT, GT38, GT39, and SNU719 are EBV-positive gastric carcinoma cell lines. PT, GT38, and GT39 cell lines were gifts from Sairenji T. (Division of Biosignaling, Department of Biomedical Sciences, Tottori University). SNU719 was kindly provided by Prof. Qian Tao (Cancer Epigenetics Laboratory, The Chinese University of Hong Kong). B95-8 is an EBV-transformed marmoset B-lymphoblastoid cell line. OB is an immortalized lymphoblastoid cell line infected by EBV. Raji is an EBV-positive BL cell line. And C666-1 is an EBV-positive NPC cell line. These four cell lines were preserved by our laboratory. All cell lines were routinely cultivated in DMEM or 1640 medium with 10% heat-inactivated fetal bovine serum (FBS), 100 U/ml penicillin, and 100 *μ*g/ml streptomycin (Invitrogen, USA) at 37°C in a humidified atmosphere of 5% CO_2_ in air [22].

Fresh and paraffin-embedded gastric carcinoma tissues were obtained from gastric carcinoma (GC) patients in Shandong Province, China. The positivity of EBV in GC tissues was determined by in situ hybridization of EBV-encoded small RNA1, as described previously [23]. 102 EBVaGCs were screened out and 43 cases were used in this study. This study was approved by the Medical Ethics Committee at the Medical College of Qingdao University, China, and informed consent was received from all patients. All the methods carried out in this article were in accordance with the approved guidelines.

### 2.2. Treatment of Cell Lines with Demethylation Agent

When cells were cultivated to a 70%–80% convergence, B95-8, Raji, GT38, GT39, and SNU719 cells were treated daily with 10 *μ*mol/L 5-aza-2′-deoxycytidine (5-Aza-CdR) (Aza, Sigma-Aldrich, USA). The untreated cells were used as control. Three days after the treatment, the cells were harvested for DNA and RNA extraction.

### 2.3. DNA Extraction

DNA was extracted from the cell lines and fresh tumor tissues using the standard method with proteinase K digestion and phenol-chloroform purification. The QIAamp DNA FFPE Tissue kit (QIAGEN GmbH, Germany) was used to extract the DNA from paraffin-embedded tumor tissues.

### 2.4. RNA Extraction and Reverse Transcription

Total RNA were extracted from cell lines using TRIzol Reagent (Invitrogen, USA) as previously [22]. The residual DNA in the RNA samples was eliminated using DNase I kit (Thermo Scientific, USA) according to the manufacturer's instructions.

1 *μ*g of total RNA was used to synthesize cDNA with reverse transcription kit (Roche, Switzerland) in a total volume of 20 *μ*l according to the manufacturer's instructions. The cDNA was stored at −20°C for use.

### 2.5. DNA Bisulfite Treatment and Methylation Analysis

Bisulfite modification of DNA was carried out as described previously [24]. The methylation status of BARF1 and BHRF1 promoters was determined using methylation-specific PCR (MSP) [22, 24]. Further, bisulfite sequencing PCR (BSP) was used to detect the methylation status of the CpG locus and to verify the MSP results as described [22, 25].

The MSP product was analyzed on a 2% agarose gel. The positive product was extracted and purified from the gel and sent to Beijing Genomics Institute (BGI) for DNA sequencing using Sanger method. In this study, we selected Raji, B95-8, GT39, and GT38 cell lines for BSP. The sequences of the primers for MSP and BSP are listed in Table 1.

### 2.6. Quantitative Real-Time PCR (qRT-PCR)

Total RNA of B95-8, Raji, GT38, GT39, and SNU719 and ten EBVaGC tissues (Q31, Q89, Q148, Q173, Q205, Q225, PL-1, Q64, Q215, and Q236) were extracted before and after 5-Aza-CdR treatment as described above. The mRNA expression of BARF1 and BHRF1 was detected by real-time qPCR according to the manufacturer's instructions of Essential DNA Green Master Faststart (Roche, Switzerland).

All reagents used for qRT-PCR were obtained from Faststart Essential DNA Green Master mixes and kits (Invitrogen, USA). The total volume for PCR was 20 *μ*l, consisting of 2×Faststart Essential DNA Green Master Mix 10 *μ*l, 0.5 *μ*l forward and reverse primers, and cDNA template 2 *μ*l and then RNase-free water was added to the total volume of 20 *μ*l. The qRT-PCR was carried out by LightCycler® 96 System (Roche, Switzerland).

Three biological replicates were performed on every specimen to reduce the experimental error, and the average values of the 3 test results were analyzed. The relative mRNA expression of BARF1 and BHRF1 was calculated by the formula 2^−ΔCt^ or 2^−ΔΔCt^.

### 2.7. Statistical Analysis

Statistical analysis of promoter methylation status of BARF1 and BHRF1 in the cell lines was performed by SPSS17.0 statistical software; Student's *t*-test was used to compare the relative expression of BARF1 mRNA and BHRF1 mRNA in EBV-positive cell lines before and after 5-Aza-CdR treatment. *P* < 0.05 was statistically significant.

The methods were according to the previous study of Li et al. [26].

## 3. Results

### 3.1. Methylation Status of BARF1p and BHRF1p in EBV-Positive Cell Lines

**Figure 1:**
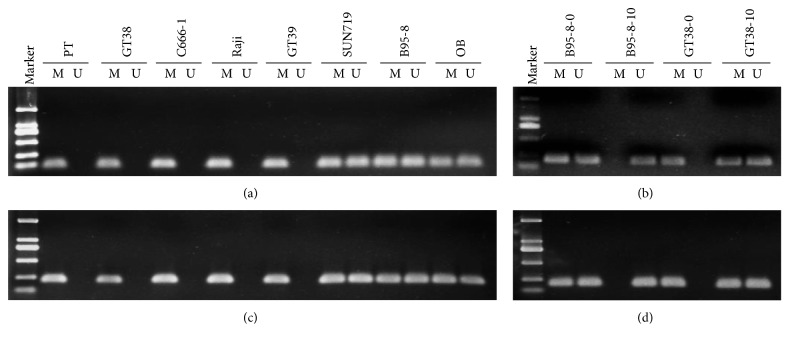
The methylation status of BARF1p and BHRF1p analyzed by MSP in EBV-positive cell lines. (a, c) MSP analysis of the methylation status of BARF1 and BHRF1 gene promoters before 5-Aza-CdR treatment in EBV-positive cell lines: PT, GT38, C666-1, Raji, GT39, SNU719, B95-8, and OB. (b, d) MSP analysis of the methylation status of BARF1 and BHRF1 in EBV-positive cell lines after 5-Aza-CdR treatment. (a, b) BARF 1p. (b, d) BHRF1p. M, methylated; U, unmethylated.

MSP was taken to evaluate the promoter methylation status of BARF1and BHRF1 in EBV-positive cell lines. BARF1 and BHRF1 promoters in PT, GT39, GT38, C666-1, and Raji were all methylated (form M), while they were form M + U (methylated and unmethylated) in SNU719, B95-8, and OB. Results were shown in Figures 1(a) and 1(c).

**Figure 2:**
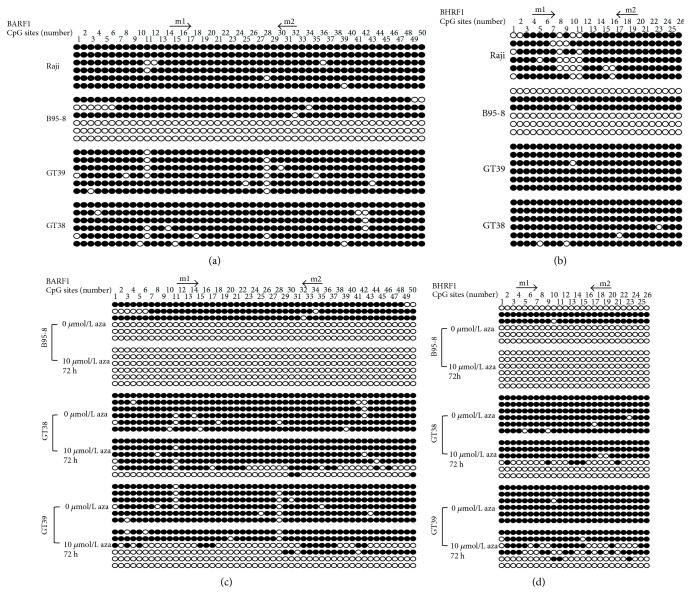
The methylation status of CpG about BARF1and BHRF1 gene promoters (BSP). Each circle is one CpG site and filled circles are methylated CpG sites. (a, b) The methylation status of CpG about BARF1 and BHRF1 gene promoters before 5-aza treatment. There are 50 and 26 CpG sites in BARF1 and BHRF1 promoters, respectively. The methylation ratio of BARF1 before 5-aza in Raji, B95-8, GT39, and GT38 was 98.0%, 46.7%, 48.3%, and 95.3%, respectively, and that of BHRF1 was 83.3%, 32.7%, 16.0%, and 97.4%, respectively. (c, d) The methylation status of CpG about BARF1and BHRF1 gene promoters before and after 5-aza treatment. After 10 *μ*mol/L 5-aza treatment, the methylation ratio of BARF1 in B95-8 and GT38 turned to 0 and 75.0% and that of BHRF1 turned to 0 and 52.6%, respectively.

To further confirm the MSP results and characterize the methylation status of BARF1 and BHRF1 in detail, we detected the methylation status of 50 and 26 CpG sites spanning BARF1 and BHRF1 promoter region by high-resolution bisulfite sequencing PCR (BSP) in Raji, B95-8, GT39, and GT38 cell lines, respectively. Purified PCR products were ligated to the pMD-18 simple vector and were transformed into the receptor bacteria DH5*α*, and then 6 positive clones were selected for Sanger sequencing. The methylation ratio was calculated by the methylated CpG sites to all the CpG sites in the 6 positive clones. Results showed that most CpG loci in the selected region of BARF1 and BHRF1 promoters were methylated in GT39, GT38, and Raji. While in B95-8, three of the six BARF1 T-A clones were nearly completely methylated, and the other three clones were unmethylated. For BHRF1, there were two clones methylated and four unmethylated in B95-8. All these were in line with their MSP results (Figures 1 and 2). For 300 CpG sites detected in BARF1, the methylated sites were 140 (46.7%), 284 (94.7%), and 286 (95.3%) in B95-8, GT39, and GT38 cells, respectively. Of all 156 BHRF1 CpG sites, the methylated ratio in B95-8, GT39, and GT38 cells was 32.7% (51/156), 99.4% (155/156), and 97.4% (152/156), respectively (Figure 2 and Table 2).

To study whether 5-Aza-CdR could demethylate the CpG methylation of the BARF1p and BHRF1p, we treated the EBV-positive cell lines, Raji, B95-8, GT39, and GT38, with 10 *μ*mol/L 5-Aza-CdR for 3 days. MSP analysis showed that the methylation status of BARF1 and BHRF1 promoters in B95-8 both changed into form U from form M + U after 5-Aza-CdR treatment, and they both changed into form M + U from form M in GT38 (Figures 1(b) and 1(d)). Concomitantly, the high-resolution BSP revealed that the methylated alleles of both BARF1p and BHRF1p became unmethylated in B95-8, the methylation ratio of the 300 and 156 CpG loci in the two genes both became 0 from 46.7% (140/300) and 32.7% (51/156), respectively. In GT39 and GT38, the unmethylated alleles of both BARF1p and BHRF1p were increased and the hypermethylation degree decreased, and the methylation ratio of GT39 in CpG island in the two genes changed to 43.0% (129/300) and 53.2% (83/156) from 94.7% (284/300) and 99.4% (155/156), while the methylation ratio of GT38 changed to 75% (225/300) and 52.6% (82/156) from 95.3% (286/300) and 97.4% (152/156), respectively (Figures 2(c) and 2(d) and Table 2). The BSP analyses were all well accordant to MSP results (Table 2).

### 3.2. BARF1 and BHRF1 Methylation in EBVaGC Tissues

**Figure 3:**
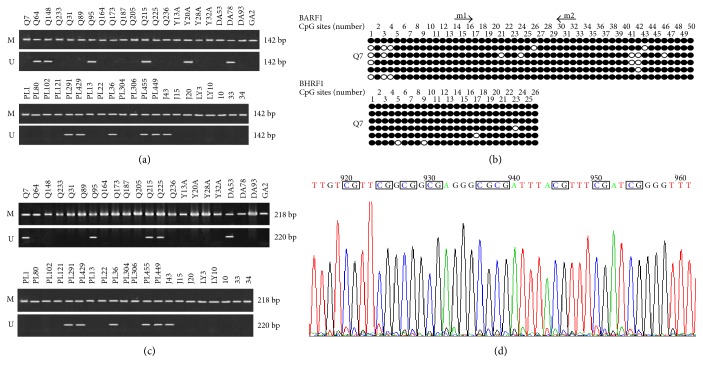
The methylation status of BARF1 and BHRF1 promoters in gastric carcinoma tissue by MSP and BSP. (a, c) The methylation status of BARF1 and BHRF1 promoters in gastric carcinoma tissue by MSP. M, methylated; U, unmethylated. (b) The methylation status of the 2 early gene promoters in gastric carcinoma tissue by BS. “Black circle” indicates methylated CpG site; “white circle” indicates unmethylated CpG site. The CpG sites in BSP region of BARF1and BHRF1 were 50 and 26. (d) Sequencing result of BARF1 gene promoter in EBV-positive gastric carcinoma cell line named GT38. The 8 CpG sites in the sequence correspond to 32–40 CpG sites in Figure 2(a).

The methylation status of BARF1 and BHRF1 was detected by MSP in 43 EBVaGC tissues. The results showed two methylation status: forms M and M + U. For BARF1, 31 cases were of form M (31/43, 72.1%) and 12 of M + U (12/43, 27.9%). For BHRF1, the cases of forms M and M + U were 36 (36/43, 83.7%) and 7 (7/43, 16.3%), respectively (Figure 3).

We further studied the detailed methylation profiles of BARF1 and BHRF1 promoters by BSP in EBVaGC tissues. Results revealed that the CpG sites of BARF1 and BHRF1 were heavily methylated in all studied samples (Figure 3(b) shows the representative specimen), which were consistent with the MSP results. The rare unmethylated sites in BARF1 were mainly CpG1, 3, and 42 of the 50 loci, while the unmethylated site of BHRF1 were scattered (Figures 3(b) and 3(d)).

### 3.3. The Expression of the BARF1 and BHRF1 mRNA in Cell Lines and Tumor Samples

**Figure 4:**
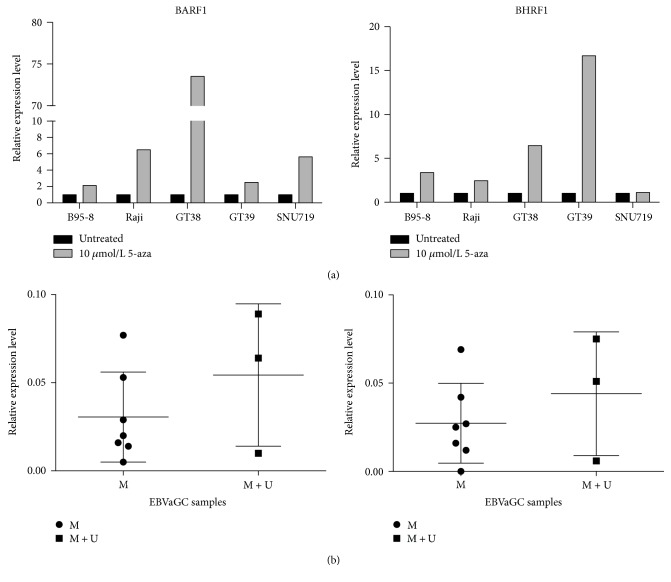
The relative expression of BARF1 and BHRF1. (a) Expression level of BARF1 and BHRF1 in the cell lines before and after treatment with demethylation reagent. Analysis used was the 2^−ΔΔCt^ method, and the expression level of untreated cells was set as 1. The expression of BARF1 in all cell lines was significantly upregulated after 5-aza treatment (*P* < 0.05); the expression difference of BHRF1 in SNU719 before and after treatment was not significant. While the difference of BHRF1 in other cell lines was significant (*p* < 0.05). (b) Expression level of BARF1 and BHRF1 in EBVaGC samples. The level showed in the figure is mean ± SD. The expression of BARF1 and BHRF1 in samples with form M + U (*n* = 3) was higher than that in the samples with form M (*n* = 7), but the difference is not significant. EBVaGC, EBV-associated gastric carcinoma. M, methylated; U, unmethylated.

The relative expression of BARF and BHRF1 mRNA to GAPDH in B95-8, Raji, GT38, GT39, and SNU719 cells was measured before and after 10 *μ*mol/L 5-Aza-CdR treatment by qRT-PCR. The relative expression of BARF and BHRF1 were calculated using 2^−ΔCt^ (Table 3) or 2^−ΔΔCt^ (Figure 4(a)). The expression of untreated cells was used as a calibrator and set as 1.

The results showed that BARF1 mRNA expression in the five EBV-positive cell lines were all significantly increased after 5-Aza-CdR treatment compared to untreated cells (Figure 4(a), Table 3). The relative BARF1 mRNA expression after 5-Aza-CdR treatment in Raji, B95-8, GT39, GT38, and SNU719 was 6.4, 2.1, 2.5, 73.1, and 5.8 times of that before treatment, respectively. And the relative expression of BHRF1 mRNA in five cell lines after 5-Aza-CdR treatment was 2.3, 3.4, 16.7, 6.5, and 1.1 times before 5-Aza-CdR treatment. Statistical analysis showed that the differential expression of BHRF1 mRNA in Raji, B95-8, GT39, and GT38 was significantly different (*P* < 0.05) (Figure 4(a) and Table 3). Ten EBVaGC tumor samples were used to analyze the expression of BARF1 and BHRF1. The expression of both genes in the samples with form M + U (*n* = 3) was higher than that in the samples with form M (*n* = 7) (Figure 4(b)). In form M + U, the expression level of BARF1 and BHRF1 was 0.054 ± 0.023 and 0.044 ± 0.02, respectively. And in form M, the relative expression level of BARF1 and BHRF1 was 0.031 ± 0.092 and 0.027 ± 0.009, respectively. However, the differences were not significant.

## 4. Discussion

BARF1 is a potent oncogene in EBV carcinomas and has a variety of important biological functions in the pathogenic and carcinogenic mechanism of EBV. BARF1 has the ability to transform the EBV-negative human B cells-Louckes, mouse fibroblast cell line BALB/c3T3, and EBV-negative lymphoma cell line Akata and immortalize human bronchial epithelial cell line-HBE cells [27–30]. It can also enhance the antiapoptotic ability of BGC823 cells [31]. BARF1 had been reported to be expressed in EBV-positive NPC and GC tissues during latency and was considered as an oncogene, parallel to the more widely studied latent membrane protein 1 (LMP1) [10, 32]. Especially in EBV-positive GC, BARF1 is expressed in the absence of LMP1, possibly functioning as an EBV oncogene in this disease and playing an important role in the occurrence and development of EBVaGCs [12, 14, 16]. In addition, BARF1 is rarely detected in protein level. In vitro study investigated that BHRF1 was quickly secreted into the culture medium and was hardly detectable in the cell [12, 33]. Expression of the BARF1 prompter during lytic replication is regulated by the immediate early proteins BRLF1 (R) and is independent of the promoter methylation status [15]. However, the methylation status of BARF1 promoter may play an important role in cancer progression during latency infection of EBV.

BHRF1 is an immediate early gene which is homologous with the Bcl-2 oncogene, encoding a protein with 25% of the AA sequence the same with Bcl-2 protein [34]. BHRF1, functionally similar to Bcl-2, could inhibit apoptosis of the B lymphocytes and epithelial cells. In vitro studies showed that BHRF1 could enhance cell resistance to apoptosis and cell death caused by many external factors, such as foreign (heterologous) virus infection, chemotherapy drugs, and rays. Dawson et al. confirmed that BHRF1 could delay the differentiation of epithelial cells by inhibiting apoptosis, which may be involved in the formation of epithelial tumor cells [35]. BHRF1 mRNA contains leading sequence of EBNA family; therefore, it could also be expressed in the latent period and transcribed by promoter Cp or Wp, while its transcription is initiated by Hp in lytic replication cycle [18]. Highly expressed BHRF1 in lytic replication stage can cause the generation of viruses by inhabiting cell apoptosis, while the low expression level in the latent period sustains the persistent infection. BHRF1 plays an important role not only in the effective replication of virus but also in the release of mature viral particles [18].

In this study, we explored the CpG methylation profiles of EBV early genes BARF1 and BHRF1 in EBV-positive cell lines and EBVaGCs tissues by MSP and BSP and further evaluated the promoter CpG methylation and their mRNA expression before and after 5-Aza-CdR treatment. We found that both BARF1p and BHRF1p were hypermethylated (form M) in EBV-positive gastric cancer cell lines PT, GT38, GT39; EBV-positive NPC cell line C666-1; and B cell lymphoma cell line Raji, along with the very low mRNA expression of BARF1 and BHRF1 by real-time qPCR. In SNU719, B95-8, and OB, BARF1p and BHRF1p were partially methylated (form M + U), and higher expression of BARF1 and BHRF1 were found in B95-8.

After demethylation by 5-Aza-CdR, the methylation of BARF1p was decreased in the cell lines and the restored expression of BARF1 mRNA was found in B95-8, Raji, GT39, GT38, and SNU719 cells. These suggested that BARF1 is methylation sensitive, which is consistent with the previous reports [21, 30]. The high methylation of BARF1 promoter and low expression of BARF1 may contribute to avoiding the immune response caused by the combination of BARF1 protein and human colony-stimulating factor (h-CSF) and to avoiding being attacked by the immune system and the maintenance of the EBV latent infection.

We found that BHRF1 promoter Hp was highly methylated in PT, GT38, GT39, C666-1, and Raji and EBV-positive gastric cancer tissues, while it was partially methylated in SNU719, B95-8, and OB. The results of MSP and BSP were consistent with the relative expression of the mRNA. The methylation of BHRF1 promoter could be demethylated by 5-Aza-CdR. Moreover, the demethylation by 5-Aza-CdR can result in the restored expression of BHRF1 mRNA in B95-8, Raji, GT39, and GT38 cells. However, the expression of BHRF1 did not increase in SNU719 cells which is form M + U. This indicated that the transcription of BHRF1 was sensitive to CpG methylation. Furthermore, in EBVaGCs tissues, BARF1p and BHRF1p were methylated as well, while their expression level in samples with form M + U was higher than that in the samples with form M in general. This funding suggested that the expression of EBV early genes BARF1 and BHRF1 was influenced by their promoter CpG site methylation.

During latent infection, EBV gene transcription is tightly regulated in EBV-associated malignances of different origins. DNA methylation is critically important for regulating gene expression. It is utilized as a host defense mechanism to suppress the expression of EBV viral genes. On the other hand, EBV infection could induce the methylation of tumor suppressor gene to repress their transcription and drive tumorigenesis [36].

Our study demonstrated that the promoters of BARF1 and BHRF1 were hypermethylated during latency infection. We found that the methylation level of BARF1 and BHRF1 gene promoter Ap and Hp varies in different cell lines. The methylation level and the expression of BARF1 and BHRF1 had a high negative correlation. BARF1 and BHRF1 are silenced by methylation in EBVaGC. However, the association of BARF1 and BHRF1 with EBVaGC carcinogenesis was still in question.

## Figures and Tables

**Table 1:** Sequences of primers for MSP, BSP, and real-time PCR in this study.

	Primers	Sequence (5′-3′)	Annealing temp (°C)	Product size (bp)
MSP	BARF1p-MF	GTTGGATTTAGTTATTTTGTCGTTC	73°C	142
	BARF1p-MR	TTATCATATAAACCTAAAACCCGTA		
	BARF1p-UF	GTTGGATTTAGTTATTTTGTTGTTTG	70°C	142
	BARF1p-UR	TTATCATATAAACCTAAAACCCATA		
	BHRF1p-MF	TTTGTATATTTGGTTAGTTGATCGA	68°C	218
	BHRF1p-MR	CGAAACGTAATACTTCCTAAAAACG		
	BHRF1p-UF	TTTGTATATTTGGTTAGTTGATTGA	66°C	220
	BHRF1p-UR	CCCAAAACATAATACTTCCTAAAAACA		
BSP	BARF1pBSP-F	GTTAGTTAGGTTGGTTAGGGTTTA	74°C	738
	BARF1pBSP-R	CTCCAATAAAAAATCAAAATAACTC		
	BHRF1pBSP-F	AGAATTTAGAGGAAGGGAATTTTATAGT	73°C	714
	BHRF1pBSP-R	ACAACCAAAACAAAAATAAAAAAAA		
qRT-PCR	BARF1-F	AGCCTCTCTGTTGCTGTTGA	59°C	130
	BARF1-R	AGTGCGTTTATTGCGACAAG		
	BHRF1-F	GTACCCTGCATCCTGTGTTG	59°C	72
	BHRF1-R	CTACAGTGTCCTCTGGCGAA		
	GAPDH-F	CTCAGACACCATGGGGAAGGTGA	58°C	450
	GAPDH-R	ATGATCTTGAGGCTGTTGTCATA		

**Table 2:** The methylation ratio of CpG loci in BARF1p and BHRF1p before and after 5-aza treatment.

Cell line	Treatment	Methylation rate	
		BARF1	BHRF1
B95-8	0 *μ*mol/L 5-aza	46.7%^∗^ (140/300)	32.7%^∗^ (51/156)
	10 *μ*mol/L 5-aza	0 (0/300)	0 (0/300)
GT39	0 *μ*mol/L 5-aza	94.7%^∗^ (284/300)	99.4%^∗^ (155/156)
	10 *μ*mol/L 5-aza	43.0% (129/300)	53.2% (83/156)
GT38	0 *μ*mol/L 5-aza	95.3%^∗^ (286/300)	97.4%^∗^ (152/156)
	10 *μ*mol/L 5-aza	75.0% (225/300)	52.6% (82/156)

^∗^The difference of methylation rate is significant (*P* < 0.05).

**Table 3:** The mRNA expression of BARF1 and BHRF1 in cell lines before and after 5-Aza-CdR treatment.

Cell line	Treatment	mRNA relative expression to GAPDH(2^−ΔCt^)	
		BARF1	BHRF1
Raji	Untreated	2.5 × 10^−7^	0.04
	10 *μ*mol/L 5-aza	1.6 × 10^−6^	0.09
	Ratio (after/before)	6.4	2.3
B95-8	Untreated	0.30	0.23
	10 *μ*mol/L 5-aza	0.64	0.78
	Ratio (after/before)	2.1	3.4
GT39	Untreated	6.4 × 10^−4^	2.4 × 10^−4^
	10 *μ*mol/L 5-aza	1.6 × 10^−3^	4.0 × 10^−3^
	Ratio (after/before)	2.5	16.7
GT38	Untreated	6.7 × 10^−3^	0.04
	10 *μ*mol/L 5-aza	0.49	0.26
	Ratio (after/before)	73.1	6.5
SNU719	Untreated	1.8 × 10^−3^	0.02
	10 *μ*mol/L 5-aza	10.4 × 10^−3^	0.022
	Ratio (after/before)	5.8	1.1
